# *Treponema pallidum* mRNA-LNP vaccine candidate encoding TP0954 induces strongly protective immunity in rabbits

**DOI:** 10.3389/fimmu.2026.1769155

**Published:** 2026-02-18

**Authors:** Zhiyu Lu, Yizhou Lu, Di Liu, Fangzhi Du, Qingyun Wu, Guoyang Liao, Yanan Wu, Rui-Li Zhang, Jian Zhou, Qianqiu Wang

**Affiliations:** 1 Hospital for Skin Diseases, Institute of Dermatology, Chinese Academy of Medical Sciences and Peking Union Medical College, Nanjing, China; 2 Department of Dermatology, The Second Affiliated Hospital of Nanjing Medical University, Nanjing, China; 3 Institute of Medical Biology, Chinese Academy of Medical Sciences and Peking Union Medical College, Kunming, China

**Keywords:** immunoprotecion, mRNA vaccine, syphilis, Tp0954, *Treponema pallidum*

## Abstract

**Introduction:**

Syphilis is a sexually or vertically transmitted disease caused by the infection of *Treponema pallidum* (*T. pallidum*). Syphilis prevalence has risen globally despite the availability of effective treatments. The development of a syphilis vaccine is crucial for controlling disease spread and severity. Over the decades, a variety of strategies have been examined including inactivated bacteria, subunit recombinant proteins and DNA vaccines, some of which showed promising results. Recent years, mRNA vaccines have become next-generationapproaches against infectious diseases.

**Methods:**

In this study, we successfully constructed a TP0954 mRNA vaccine and confirmed the immunogenicity of the mRNA vaccine in BALB/c mice and New Zealand White (NZW) rabbits. Then the protective immunity was assessed in immunized NZW rabbits.

**Results:**

Our mRNA vaccine elicited humoral and cellular immunological responses both in BALB/c mice and NZW rabbits. Moreover, TP0954 mRNA vaccine was more effective in attenuating lesion development compared with TP0954 protein vaccine. Similarly, the *T. pallidum* burdens at the challenge sites and distal organs in rabbits immunized with TP0954 mRNA vaccine were lower compared with TP0954 recombinant protein vaccine.

**Conclusion:**

Therefore, we successfully constructed a novel mRNA vaccine targeting TP0954 for syphilis and found superior immune protective effects compared with TP0954 recombinant protein vaccine, further confirming that TP0954 is a promising vaccine candidate for syphilis.

## Introduction

Syphilis is a sexually and vertically transmitted chronic disease caused by the spirochete *Treponema pallidum* subsp. *pallidum* (*T. pallidum*). Although syphilis can be easily diagnosed and treated with penicillin G, it continues to be a significant global health problem especially in low-income countries (1, 2). Syphilis diagnoses have increased worldwide over the past several decades. From 2019 to 2023, the overall number of syphilis cases in the United States increased by 61%. Of note, diagnoses among females increasing by 112% and cases of congenital syphilis increasing by 106% (3). In China, Syphilis incidence also exhibited an overall increasing trend. A total of 5,527,399 syphilis cases were reported, with an average annual prevalence of 25 per 100,000 population from 2004 to 2019 in China (4). Therefore, there is an urgent need to develop an effective vaccine against syphilis. Several syphilis vaccine candidates have been tested, such as inactivated bacteria and subunit recombinant proteins, some of which showed partially protective while the others showed no protection (5).

In the prevention and treatment of infectious diseases, mRNA vaccines exhibit substantial promise recent years, primarily due to low risk of insertional mutagenesis, high potency, potential for low-cost and high-efficiency manufacture (6). During the COVID-19 pandemic, a variety of vaccine platforms were developed and authorized for human administration (7). Among these, COVID-19 mRNA vaccines demonstrated notable efficacy in disease prevention, drawing significant public attention (8). This was the first time that an mRNA vaccine received license for human use. The successful application of mRNA vaccines against COVID-19 has not only further validated this platform but also unlocked the full potential of mRNA vaccines in infectious disease prevention.

Unlike virus, bacterial proteins encoded by mRNA may be poorly translated in mammalian cells. In addition, the intracellular trafficking, processing, and post-translational modifications of these heterologous proteins can substantially impact their stability and immunogenicity (9). To optimize antigen expression of bacterial proteins in mammalian cells, several approaches such as the use of trafficking, sequence optimization and bioinformatic tools have been developed (9, 10). Despite existing limitations, an increasing number of studies explored the use of mRNA vaccines for bacteria and showed promising results (11–15). Moreover, mRNA vaccines for *Y. pestis*, *L. monocytogenes*, *S. pyogenes* and *S. agalactiae*, *P. aeruginosa* and *S*. *typhimurium* were pre-clinically evaluated, and mRNA vaccines for *B. burgdorferi* and *M. tuberculosis* were under clinical evaluation, indicating that bacterial diseases have become a desired target for mRNA vaccine development (9).

Similarly, *T. pallidum* is a Gram-negative bacterium due to the double membrane structure. However, the structure, composition, and physical properties of its outer membrane (OM) are distinctly different from those of typical Gram-negative bacteria (16). The poor protein content of OM is a key feature of *T. pallidum*, contributing to the poor immunogenicity of this pathogen (5). Among the reported outer membrane proteins (OMPs), TP0954 is a well-characterized surface lipoprotein adhesin of T. pallidum that mediates bacterial attachment to epithelial, endothelial, neuronal, and placental cells. This specific placental binding capability makes it a key target for preventing congenital syphilis. Besides, TP0954 is highly conserved among *T. pallidum* strains, ensuring broad efficacy against various clinical isolates. Additionally, immunization with recombinant TP0954 (rTP0954) induced robust humoral and cellular immune responses in rabbits, It could delay lesion development, reduce bacterial dissemination to distal tissues, and confer sterile immunity in a rabbit model of experimental syphilis (17). To further examine the feasibility of this mRNA vaccine in *T. pallidum* infected model, we developed an mRNA vaccine targeting on TP0954, evaluated the antigen-specific humoral and cellular responses in mice and rabbits, and further assessed protection compared with TP0954 protein vaccine.

## Methods

### mRNA synthesis

The mRNA construct was designed based on structural architecture of the Moderna mRNA-1273 which has been shown to be highly effective in ensuring stability, protein expression and immunogenicity. Specifically, we incorporated the 5’ untranslated region (UTR), the 3’ UTR, and the poly(A) tail in the mRNA sequence for the TP0954 antigen. Plasmids corresponding to the full sequence of TP0954 was amplified using E. coli Stabl3 (ThermoFisher Scientific, Waltham, MA, USA). Following plasmid extraction, linearized templates were generated via digestion with the BspQI restriction enzyme and purified using DNA magnetic beads (Vazyme, Nanjing, China). Subsequently, *in vitro* transcription (IVT) was performed to synthesize messenger RNA (mRNA), using T7 RNA polymerase (Vazyme, Nanjing, China), CleanCap (Syngenbio, Nanjing, China), and deoxyribonucleoside triphosphates (dNTPs)-where uridine triphosphate (UTP) was substituted with m¹ψ-5′ triphosphate (Syngenbio, Nanjing, China). After completion of the IVT reaction, the synthesized mRNA was purified using RNA magnetic beads (Vazyme, Nanjing, China). An ultraviolet spectrophotometer quantified mRNA concentration and formaldehyde-denaturing agarose gel electrophoresis assessed mRNA integrity and purity.

### Cell culture, transfection and expression of TP0954 mRNA by Western blot

HEK293T cells were cultured in DMEM supplemented with 10% fetal bovine serum and 100 U/mL penicillin–streptomycin at 37°C in a humidified 5% CO_2_ atmosphere. Cells were transfected with 2 µg mRNA using Lipofectamine 3000 and cultured for 48 h. Cells were then harvested, washed with PBS, and lysed in RIPA buffer supplemented with 1 mM PMSF. Total proteins were separated on 4-20% Bis-Tris gradient gels and transferred to PVDF membranes. After blocking, membranes were incubated with primary antibodies overnight at 4°C, followed by HRP-conjugated secondary antibodies. Protein bands were visualized using chemiluminescence and imaged with a ChemiDoc MP Imaging System.

### LNP encapsulation

The prepared mRNA solutions were encapsulated in lipid nanoparticles (LNPs). mRNA solutions were formulated at a concentration of 200 μg/mL in 25 mM sodium acetate buffer (pH 5.5). The LNP formulation comprised four components: cationic lipid, phosphatidylcholine, cholesterol, and polyethylene glycol (PEG)-lipid, at a molar ratio of 50:10:38.5:1.5, which were dissolved in anhydrous ethanol. mRNA and LNP components were mixed at a 3:1 flow ratio using a microfluidic device to form an mRNA-LNP mixture. Subsequently, ethanol was removed and replaced with 25 mM Tris-HCl buffer (pH 7.5) using a 100 kDa ultrafiltration centrifugal filter, and the solution was concentrated to produce the mRNA-LNP vaccine. Encapsulation efficiency was determined using the Quant-iT™ RiboGreen RNA Kit (ThermoFisher Scientific, Waltham, MA, USA), while particle size and polydispersity index were measured with a Nanoparticle Size and Zeta Potential Analyzer (Malvern Panalytical, Malvern, UK).

### Mouse immunization

Female BALB/c mice (6–8 weeks old, specific-pathogen-free) were used in this study, supplied by GemPharmatech Co., Ltd. (Nanjing, China). Four groups were established by random allocation, with 5 mice per group. Mice were intramuscularly injected with TP0954 mRNA vaccine at different doses (1 μg, 5 μg, 10 μg) into the hind limb. For the LNP control group, mice received an equivalent volume of LNPs alone. Primary immunization was administered on day 0, with a booster dose given on day 14. Blood samples were collected via cardiac puncture on day 14 and 28. On day 28, following anesthesia, spleens were harvested. This animal study was approved by the Ethics Committee of the Hospital of Dermatology, Chinese Academy of Medical Sciences.

### Rabbit immunization

Male New Zealand White (NZW) rabbits (8–13 weeks old, weighing 2.5–3.0 kg) were used in this study. The rabbits were randomly assigned to three groups with three animals per group. Animals in the TP0954 mRNA vaccine immunized group received an intramuscular injection of 5 μg dose of TP0954 mRNA vaccine into the hind limb. Animals in the recombinant TP0954 protein immunized group received an intramuscular injection of 100 μg dose of purified TP0954 protein mixed with an equal volume of TiterMax Gold Adjuvant. The 100 µg dose of protein was chosen based on precedent in rabbit models of syphilis vaccination to ensure sufficient antigen exposure and reproducible immune responses (18). The LNP group was administered an equivalent volume of LNPs. Primary immunization was performed on day 0, with a booster immunization given on day 14. Blood samples were collected from the ear veins on days 14 and 28.

### *T. pallidum* challenge procedure

Two weeks following the booster immunization, dorsal fur of all rabbits was shaved, and the exposed skin was disinfected with 75% ethanol. Each rabbit was intradermally challenged at 8 distinct sites: at each site, 0.1 mL of a suspension containing 1×10^6^ freshly isolated *T. pallidum* Nichols strain per mL (prepared in 0.9% saline) was administered. *T. pallidum* Nichols strain was propagated in healthy male NZW rabbits via intratesticular inoculation and harvested as previously described by Lukehart et al. (19) The development of lesions at the challenge sites was recorded and photographed daily. Additionally, the diameter of each lesion was measured daily. Blood samples used for serological test were collected from the ear veins on day 0, 7, 14, 21 post challenge. 21 days post *T. pallidum* challenge, rabbits from each group were euthanized, the lesions and organs were harvested for subsequent experiments.

### Safety of mRNA vaccine

To monitor short-term systemic responses and safety signals after immunization, consistent with international guidance on nonclinical evaluation of vaccines. Rectal temperature and body weight of mice were measured for 7 days after primary immunization. Blood sample was collected in each group at 4 weeks following the first immunization. Serum was then separated after centrifugation. Serum biochemical indicators were automatically measured using an automatic biochemistry analyzer (Chemray 800, China). 2 weeks post the final immunization, all mice were euthanized, the organs were harvested, fixed, embedded in paraffin, and sectioned for histopathological analysis. Following conventional hematoxylin and eosin (H&E) staining, the tissue sections were examined under a light microscope.

### Analysis of specific antibody levels

To evaluate the specific antibody response, blood sample was collected in each group at 2 and 4 weeks following the primary immunization. The blood was then centrifuged in order to separate the serum. The specific antibody levels were determined by indirect ELISA. The 96-well plates were coated with 1 ug/mL purified recombinant TP0954 protein. Excess antigen was washed off with PBST. Then 200 mL of blocking buffer (1g BSA dissolved in 50mL PBS) was added and incubated at 37 °C for 2 hours. Then the wells were washed three times with PBST. Rabbit serum was two-fold serially diluted starting from a dilution of 1:100. After 1 hour of incubation at 37 °C, the wells were washed three times with PBST again. Horseradish peroxidase (HRP)-conjugated goat anti-mouse IgG (1:10,000) or horseradish peroxidase (HRP)-conjugated goat anti-rabbit IgG (1:10,000) was subsequently added to the wells and incubated at 37 °C for 1 hour. The plates were washed three times with wash buffer, and 100 mL of TMB peroxidase substrate was added per well and incubated at 37 °C for 15 minutes. Then 100 mL stop solution was added to stop the color reaction. The absorbance at 450 nm (A450 value) was detected with a microplate reader ((Infinite 200 Pro, Switzerland)). The endpoint titer was considered to be the last serum dilution with readings higher than the 2.1-fold of the negative controls.

### Preparation of splenocyte suspension

Two weeks post the final immunization, mice were euthanized, and their spleens were harvested for preparation of splenic cell suspensions. 21 days post *T. pallidum* challenge, rabbits were all euthanized and spleens were harvested. Each spleen was homogenized through a 200-mesh cell strainer to obtain a splenic cell suspension. The suspension was centrifuged at 2000 rpm for 10 minutes. Following centrifugation, red blood cell (RBC) lysate was added to lyse RBCs, and the mixture was incubated for 5 minutes. Phosphate-buffered saline (PBS) was then added to terminate the lysis reaction. After another centrifugation step (2000 rpm, 5 minutes), PBS was added to wash the splenic cell suspension. A third centrifugation (2000 rpm, 5 minutes) was performed, and the resulting splenocytes were resuspended in RPMI-1640 medium supplemented with 10% fetal bovine serum (FBS), penicillin (100 U/mL), and streptomycin (100 μg/mL) for subsequent detection assays.

### Measurement of mouse and rabbit IFN-γ, TNF-α and IL-2 secretion

Mouse and rabbit splenic lymphocytes collected as described above were seeded in 6-well culture plates at a density of 1×10^7^ cells per mL. Each well was treated with 10 μg/mL recombinant TP0954 protein, with PBS serving as the blank control; the plates were then incubated at 37 °C for 24 hours. After incubation, cell culture supernatants were harvested to determine the secretion levels of interferon-γ (IFN-γ), tumor necrosis factor-α (TNF-α), and interleukin-2 (IL-2) from mouse and rabbit splenocytes. Assays were performed according to the manufacturer’s instructions for the respective ELISA kits (Elabscience, China; Ruisaiqi Biotechnology, China). The absorbance at 450 nm was measured using a microplate reader (Infinite 200 Pro, Switzerland).

### Extraction of *T. pallidum* DNA and quantitative real-time PCR

DNAs were extracted from tissues of *T. pallidum*-infected rabbits by using a TIANamp Genomic DNA Kit (Tiangen, Beijing, China) following the manufacturer’s protocol. PCR amplification was performed by using a LightCycle 96 apparatus (Roche, Basel, Switzerland). Quantitative analysis of *T. pallidum* gDNA and rabbit gDNA was achieved by using primers targeting *T. pallidum* DNA Polymerase I (*polA*) gene and rabbit collagenase-1 precursor (*MMP-1*) gene, respectively. The sequences of the primers are given as followings: *polA* Sense: 5’-TACGGTGCAAGTGCTCAGAC-3’, Antisense: 5’-CAGGCACATTGTCGGAGGAA-3’; *MMP-1* Sense: 5’-TTGCTTCTTCACACCAGAATGCTGT-3’, Antisense: 5’-GCGTGATCAGGCACTATGTAGCAAT-3’. The primers for quantitative real-time PCR (qPCR) amplification were provided by Genscript Biotech (Nanjing, China). As directed by the manufacturer, qPCRamplifications were performed in 20 ul Taq Pro Universal SYBR QPCR Master Mix (Vazyme, Nanjing, China) reaction mixture. Standard curves were generated for *polA* and *MMP-1* by using 10-fold dilutions in series from 10^7^ to 10^1^ reproductions of *T. pallidum* gDNA and 2-fold dilutions in series of rabbit gDNA from 200 to 1.56 ng/ul. The following conditions were used for the PCR of *polA* and *MMP-1*: 95 °C for 5 min, 40 cycles of 95 °C for 10 sec, 60 °C for 30 sec. Following is an analysis of melt-curves: 95 °C for 10 sec, 65 °C for 60 sec, and 97 °C for 1 sec.

### Serological test

On day 0, 7, 14, 21 post *T. pallidum* challenge, blood was collected from every rabbit and serum was separated. *T. pallidum* particle agglutination assay (TPPA) and rapid plasma reagin test (RPR) were then conducted according to the instruction of TPPA Detection Kit (FUJIREBIO INC, Tokyo, Japan) and RPR Diagnostic Kit (KEHUA BIO-ENGINEERING, Shanghai, China) respectively.

### Histopathology

On day 21 post-challenge, skin lesions from each rabbit were excised, and a 6-mm punch biopsy was used to obtain standardized tissue subsamples from each lesion. These cutaneous lesion specimens were fixed, embedded in paraffin, and sectioned for histopathological analysis. Following conventional hematoxylin and eosin (H&E) staining, the tissue sections were examined under a light microscope to observe and assess the degree of inflammatory cell infiltration.

### Statistical analysis

Statistical analysis was performed with GraphPad Prism 8.0 software. Data were presented as Means ± SDs. The following tests were used to assess significance: two-tailed unpaired Student’s *t*-test between two comparisons; one-way ANOVA test and Chi-square Test of Independence for multiple comparisons. A significance level of * p < 0.05, ** p < 0.01, and *** p < 0.001 is considered to indicate significant differences in the statistical results.

## Results

### Construction and expression of TP0954 mRNA vaccine

**Figure 1:**
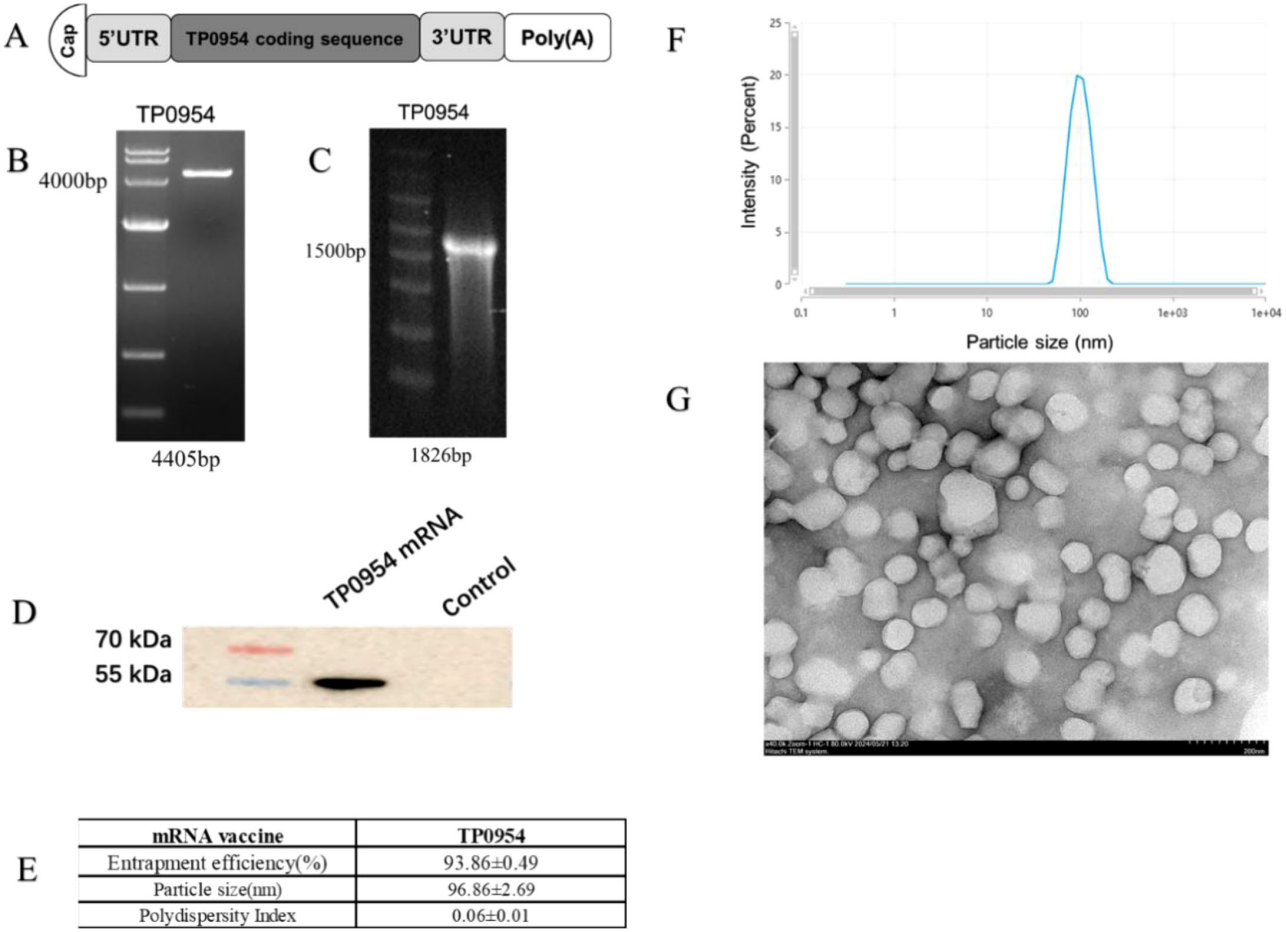
*In vitro* synthesis, characterization and expression of TP0954 mRNA vaccine. **(A)** The schematic illustration of TP0954 mRNA vaccine constructs. The mRNA constructs consist of 5’cap followed by 5’UTR, TP0954 mRNA coding sequence, 3’UTR, and poly(A) tail. **(B)** Detection of the size and integrity of linearized template was assessed by agarose gel electrophoresis. **(C)** Formaldehyde-denatured agarose gel electrophoresis assessed the integrity and size of mRNA. **(D)** Expression of TP0954 protein was achieved in HEK293T cells transfected using Lipofectamine3000 for 48h. **(E)** Physicochemical properties of TP0954 mRNA vaccine are presented as mean *±* SD. **(F)** The size distribution of LNPs was measured by a Malvern particle size instrument. **(G)** Representative TEM image illustrates the morphology of mRNA vaccines, with a scale bar of 200 nm.

The full sequence of TP0954 protein was selected as the antigenic targets for mRNA synthesis. The mRNA was constructed by integrating the coding sequence with 5’ cap, 5’UTR, 3’UTR and a poly(A) tail (**Figure 1**). The sequence of TP0954 mRNA was provided in **Supplementary Table 1**. Then, it was cloned into pUC57 plasmid. Following plasmid amplification and extraction, linearized template was generated via digestion with the BspQI restriction enzyme (**Figure 1**). High purity mRNA for vaccine purpose was obtained using *in vitro* transcription from linearized DNA (**Figure 1**). Western blot analysis confirmed the expression of a 54 kDa fragment, indicating successful translation and expression of TP0954 protein (**Figure 1**). TP0954 mRNA was encapsulated in LNPs, with an average size of 96.86 nm (**Figure 1**), and polymer dispersity index (PDI) value of LNP showed good stability of the LNP dispersion system (**Figure 1**). The encapsulation efficiency of mRNA-LNP vaccine was higher than 90% (**Figure 1**). Transmission Electron Microscope (TEM) showed spherical nanoparticles, and no obvious particle aggregation was observed (**Figure 1**).

### The mRNA vaccine showed good safety

**Figure 2:**
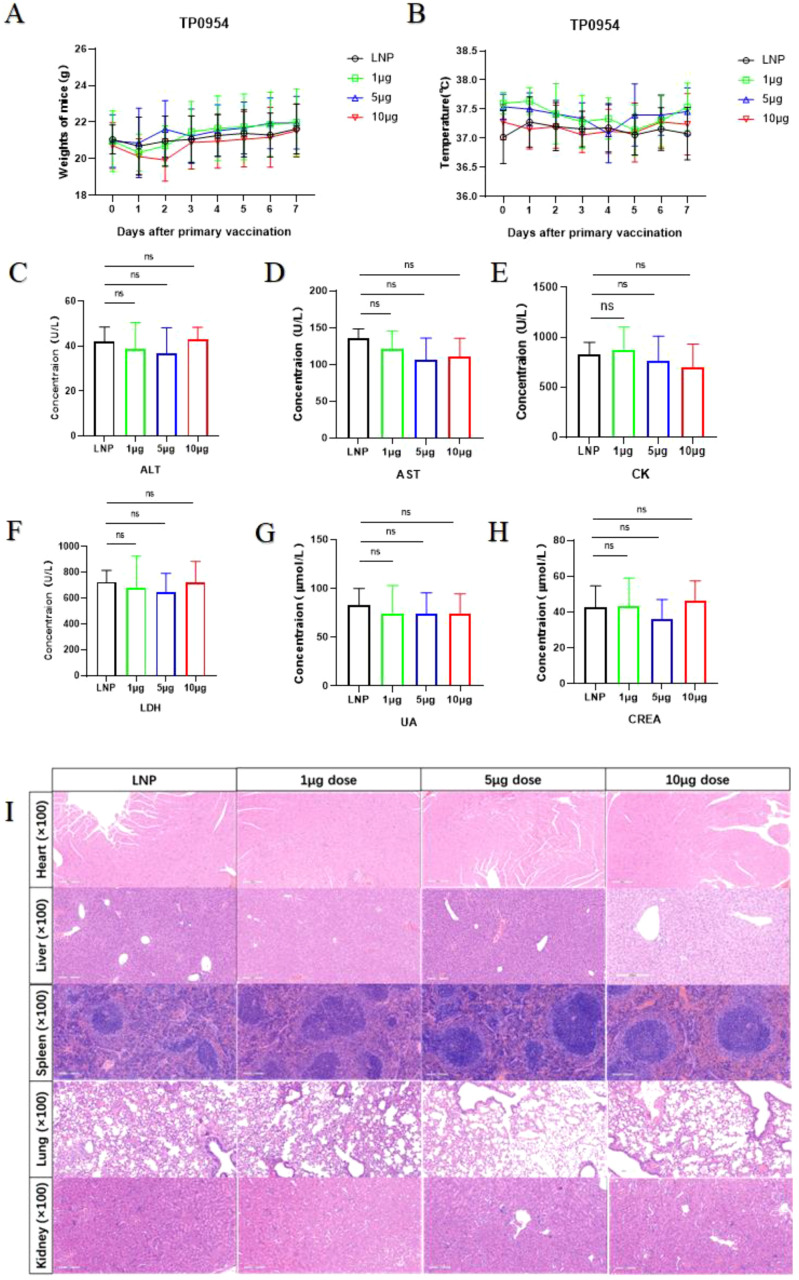
Safety examination of TP0954 mRNA vaccine. **(A)** The anal temperature of immunized mice was monitored for 7 days after the primary immunization. **(B)** The body weight of immunized mice was monitored for 7 days after the primary immunization. **(C-H)** Assay of serum biochemical indices in immunized mice. ALT, Alanine Aminotransferase. AST, Aspartate Aminotransferase. CK, Creatine Kinase. LDH, Lactate Dehydrogenase. CREA, Creatinine. UA, Uric Acid (ns, not significant). **(I)** Two weeks post boost immunization, heart, liver, spleen, lung, and kidney tissues were harvested, stained with hematoxylin and eosin (HE), and observed for morphological changes under 100×magnification, with a scale bar of 200 nm.

To preliminarily evaluate the *in vivo* toxicity of mRNA vaccine, the body temperature and body weight of immunized mice were monitored for 7 days after the primary immunization. There were no significant differences in body temperature and body weight after vaccination between immunized mice and LNP immunized mice (**Figure 2**). This observation indicated that mRNA vaccine may have good safety. Based on blood biochemical indicators, we further evaluated the functions of the heart, liver, and kidneys. Among them, creatine kinase (CK) and lactate dehydrogenase (LDH) are indicators of cardiac function; Alanine aminotransferase (ALT) and aspartate aminotransferase (AST) are indicators of liver function; Creatinine (CREA) and uric acid (UA) are indicators of renal function. As shown in (**Figure 2**), there were no significant differences in cardiac function, liver function, and renal function between mRNA vaccine immunized mice and LNP immunized mice, and all data were within the normal range. Compared with the control group, there were no obvious pathological changes in heart, liver, spleen, lung and kidney in immunized mice (**Figure 2**). Therefore, our mRNA vaccine showed good safety.

### Immunization of mRNA vaccine elicited antigen-specific humoral and T-cell immune responses in BALB/c mice

**Figure 3:**
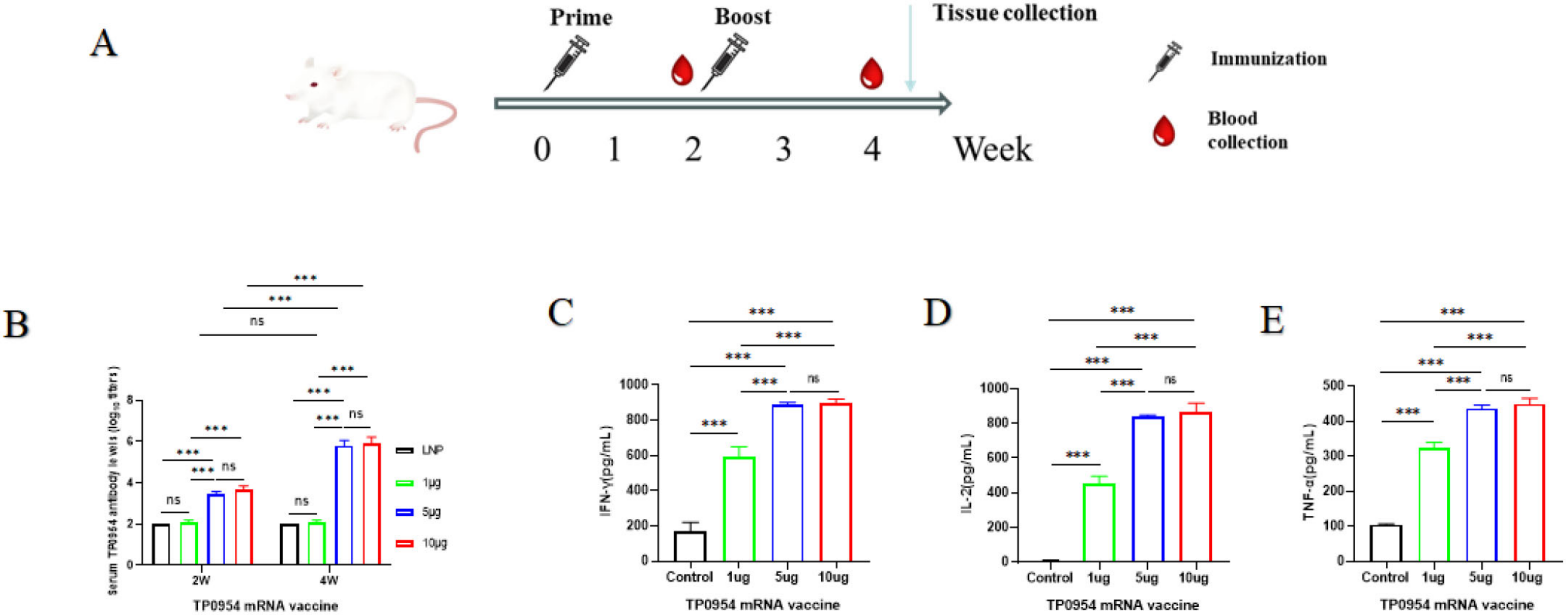
Humoral and cellular immune response of BALB/c mice activated by the immunization with TP0954 mRNA vaccine. **(A)** Immunization and sample collection timeline. Mice were intramuscularly injected with TP0954 mRNA vaccines at different doses (1 μg, 5 μg, 10 μg) into the hind limb. For the LNP control group, mice received an equivalent volume of LNPs alone. Primary immunization was administered on day 0, with a booster dose given on day 14. Blood was collected on day 14 and day 28. Two weeks post the final immunization, splenic lymphocytes were isolated. **(B)** IgG antibody levels were determined by indirect ELISA. To determine antibody concentrations (titers), two-fold serial dilutions of serum were made and the endpoint titer was considered to be the last serum dilution with readings higher than the 2.1-fold of the negative controls. **(C-E)** The levels of IFN-γIL-2 and TNF-α by ELISA in splenocytes stimulated with 10 μg recombinant TP0954 protein, with PBS stimulation as blank control. Results are expressed as the means ± SDs. Each set of data is based on measurements derived from five mice. (ns, not significant. ***P < 0.001).

To determine the immune response induced by TP0954 mRNA vaccine, groups of BALB/c mice (n = 5) were immunized intramuscularly twice with 1, 5 or 10 µg mRNA vaccine with empty LNP immunization as control. The interval between every injection was two weeks. The blood samples were collected at week 2 and week 4 (**Figure 3**). The antigen-specific antibodies were detected by indirect ELISA. As shown in **Figure 3**, 1 µg dose of TP0954 mRNA vaccine did not induce significant antigen-specific humoral immune responses. After intramuscular injection with 5 µg dose of TP0954 mRNA vaccine, the serum IgG titers significantly increased after initial immunization, and were further elevated after the second immunization. Moreover, immunization with 5 µg dose of TP0954 mRNA vaccine could induce almost the same level of IgG titer as 10 µg induced. Therefore, 5µg dose of mRNA vaccine might be the optimal dose to elicit antigen-specific humoral immune responses. IFN-γ, TNF-α and IL-2 are critical Th1-related cytokines, which is essential for early clearance of *T. pallidum* in lesions. Therefore, we also examined the levels of IFN-γ, TNF-α and IL-2 produced by mouse splenic cells by ELISA (**Figure 3**). The increased levels of IFN-γTNF-α and IL-2 induced by 5 and 10 µg dose of TP0954 mRNA vaccine were higher than those of 1 µg dose. Therefore, 5 µg dose of TP0954 mRNA vaccine was sufficient to elicit Th1-related immune responses in BALB/c mice.

### Immunization of mRNA vaccine elicited antigen-specific humoral and T-cell immune responses in NZW rabbits

**Figure 4:**
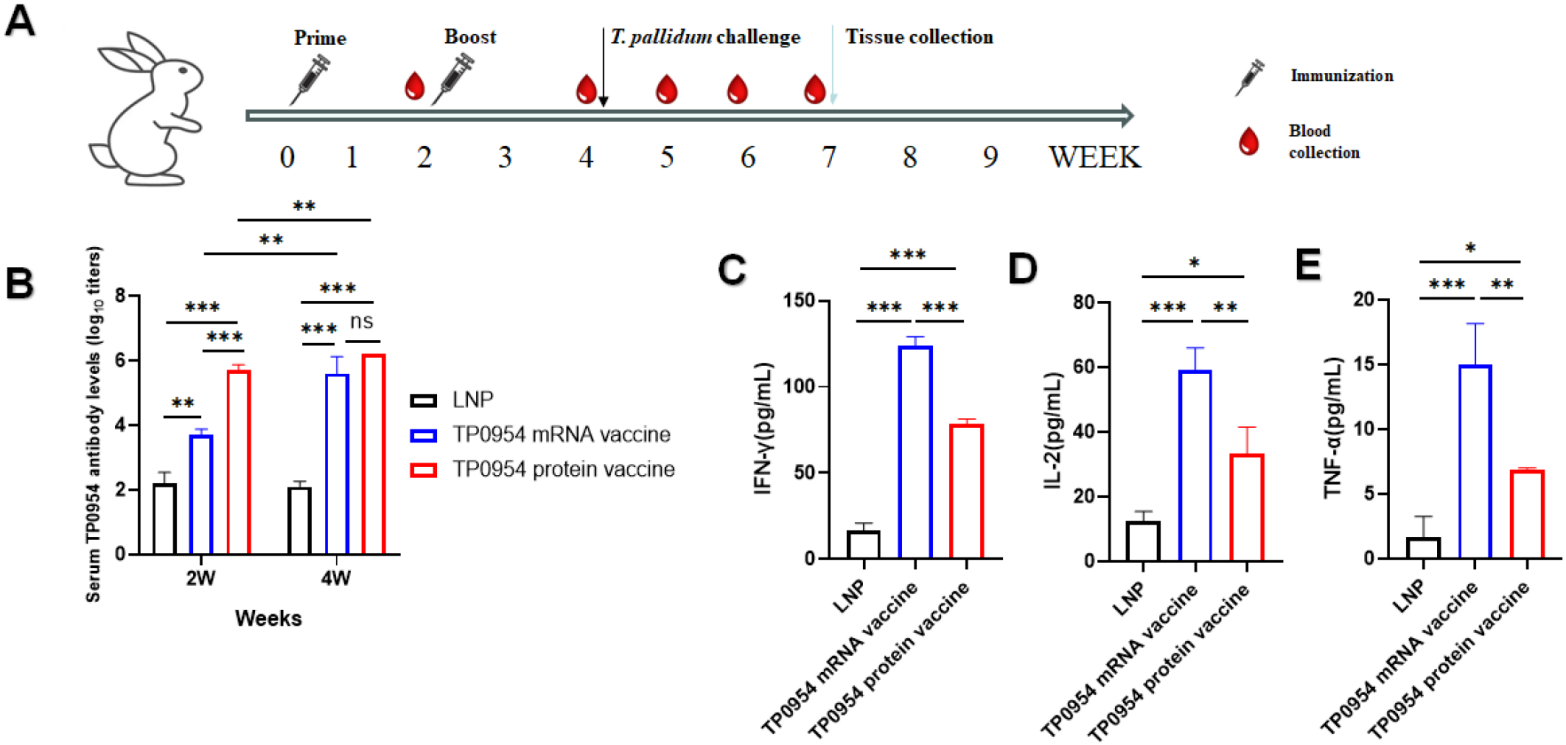
Humoral and cellular immune response of NEW rabbits activated by the immunization with 5 ug dose of TP0954 mRNA vaccine and 100 ug dose of TP0954 protein vaccine. **(A)** Immunization, *T. pallidum* challenge and sample collection timeline Each rabbit was immunized once every two weeks for two times. Blood was collected on day 14 and day 28. Two weeks post the final immunization, each rabbit was intradermally challenged at 8 distinct sites: at each site, 0.1 mL of a suspension containing 1×10^6^ freshly isolated *T. pallidum* Nichols strain per mL (prepared in 0.9% saline) was administered. 21 days post challenge, all rabbits were euthanized and their skin lesions and organs were harvested. **(B)** The serum IgG titers against TP0954 were determined by indirect ELISA at weeks 2 and 4 post primary immunization. To determine antibody concentrations (titers), two-fold serial dilutions of serum were made and the endpoint titer was considered to be the last serum dilution with readings higher than the 2.1-fold of the negative controls. **(C-E)** The levels of IFN-γIL-2 and TNF-α by ELISA in splenocytes stimulated with 10 μg recombinant TP0954 protein, with PBS stimulation as blank control. Results are expressed as the means ± SDs. Each set of data is based on measurements derived from three rabbits. (ns, not significant. *P < 0.05, **P < 0.01, ***P < 0.001).

Based on the results of mRNA vaccine elicited antigen-specific humoral and T-cell immune responses in BALB/c mice, 5 µg dose of TP0954 mRNA vaccine was used for further immunization in rabbits. To determine whether TP0954 mRNA vaccine was able to induce specific antibody response in rabbits, blood sample was collected in each group at 2 and 4 weeks following the first immunization (**Figure 4**). Then we performed serial two-fold dilutions of serum to detect specific antibody titers. As shown in **Figure 4**, TP0954 mRNA vaccine induced higher titers of specific antibody starting at week 2 compared with LNP-immunized rabbits, but lower titers compared with TP0954 protein vaccine immunized rabbits. Moreover, antibody levels in animals immunized TP0954 mRNA and protein vaccine after the second immunization were similarly high and were higher compared with primary immunization. These results indicated that TP0954 mRNA vaccine was able to induce strong antigen-specific humoral response. 21 days post *T. pallidum* challenge, rabbits were all euthanized and spleens were harvested. With stimulation with 10 μg/mL recombinant TP0954 protein for 24 hours, after incubation, cell culture supernatants were harvested to determine the secretion levels of IFN-γ, TNF-α and IL-2. Compared to that of rabbits immunized with LNP, the production of IFN-γ, TNF-α and IL-2 was significantly increased in the rabbits immunized with TP0954 protein vaccine. And TP0954 mRNA vaccine induced significantly higher level of IFN-γ, TNF-α and IL-2 compared to TP0954 protein vaccine, indicating that TP0954 mRNA vaccine could induce stronger Th1- related immune responses in NZW rabbits (**Figure 4**).

### TP0954 mRNA vaccine immunization attenuated lesion development

**Figure 5:**
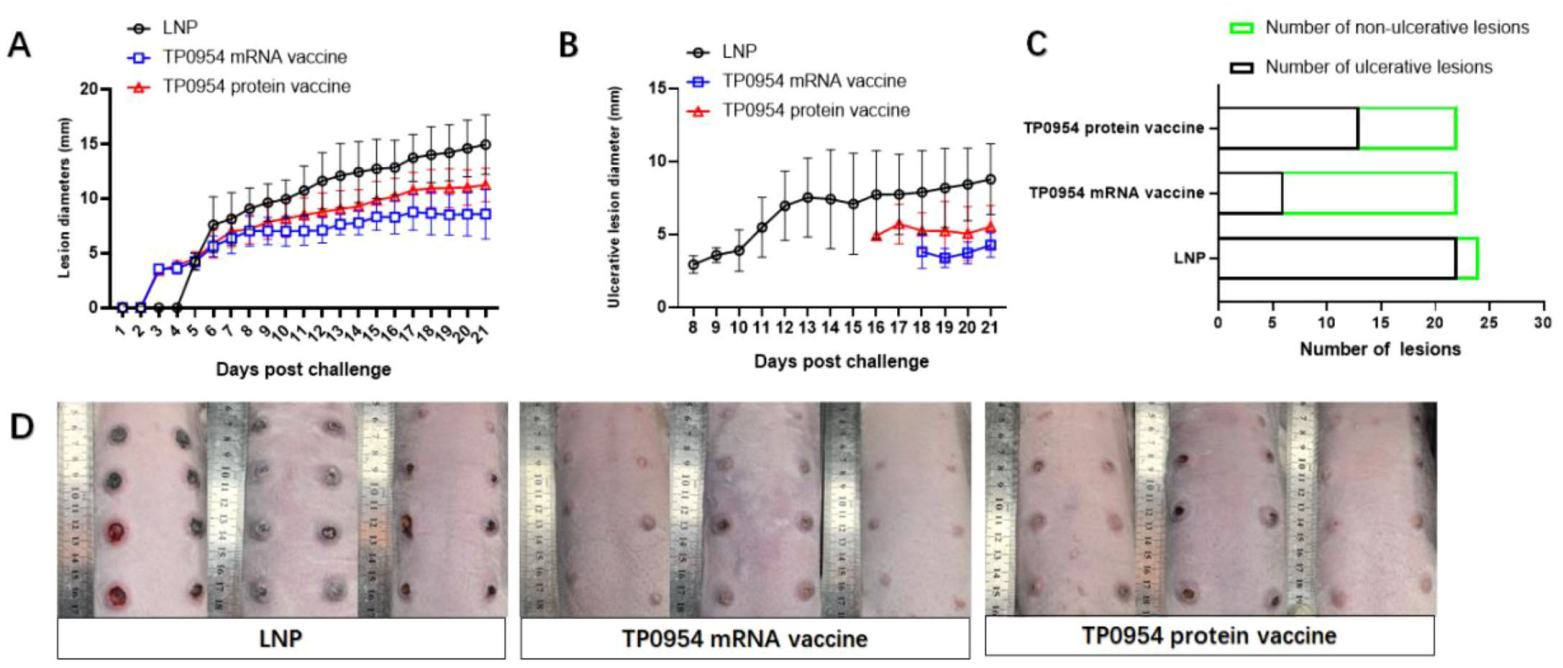
Immunization with TP0954 mRNA vaccine could attenuated lesion development. **(A)** The diameter of each induration was measured daily over 21 days post-intradermal challenge with *T. pallidum*. Results are expressed as the means ± SDs. **(B)** The diameter of each ulceration was measured daily over 21 days post-intradermal challenge with *T. pallidum*. Results are expressed as the means ± SDs. **(C)** The number of total indurations and ulcerations on 21 days post-intradermal challenge with *T. pallidum*. **(D)** Lesions on the backs of the rabbits on 21 days post-intradermal challenge with *T. pallidum*.

Two weeks after the booster immunization, each rabbit was challenged intradermally with 0.1 ml of 1×10^6^ freshly isolated *T. pallidum* Nichols strain per ml in 0.9% saline at each of 8 sites. During the subsequent 21 days, induration and ulceration were monitored daily for diameters. As shown in **Figure 5**, obvious indurations were present on rabbits by day 5, 3 and 3 in LNP group, TP0954 mRNA vaccine group and TP0954 protein vaccine group, respectively. Both rabbits immunized with TP0954 mRNA and protein vaccine showed delayed lesion development compared to the LNP group. On day 21, the average diameter of indurations in LNP group, TP0954 mRNA vaccine group, TP0954 protein vaccine group was 14.95 ± 2.72mm, 8.57 ± 2.27 mm, 11.24 ± 1.53 mm, respectively (**Figure 5**). Moreover, ulcerations presented on day 8 in LNP group, day 18 in TP0954 mRNA vaccine group and day 16 in TP0954 protein vaccine group (**Figure 5**). In addition, as shown in **Figure 5**, on day 21, the numbers of ulcerations were significantly less in TP0954 mRNA vaccine group (6/22) and TP0954 protein vaccine group (13/22) than LNP group (22/24). Similarly, the average size of ulcerations was also smaller in TP0954 mRNA vaccine group (4.25 ± 0.83mm) and TP0954 protein vaccine group (5.52 ± 1.45mm) than LNP group (8.77 ± 2.43mm). Lesions on the backs of the rabbits on 21 days post-intradermal challenge with *T. pallidum* were presented in **Figure 5** (Pictures of high-definition, un-magnified and whole-field original image of the skin were presented in **Supplementary Figure 1**). All these results indicated that immunization with TP0954 mRNA vaccine could be able to significantly delay the development of both indurations and ulcerations, and showed better efficiency compared to TP0954 protein vaccine.

### TP0954 mRNA vaccine immunization inhibited *T. pallidum* dissemination

**Figure 6:**
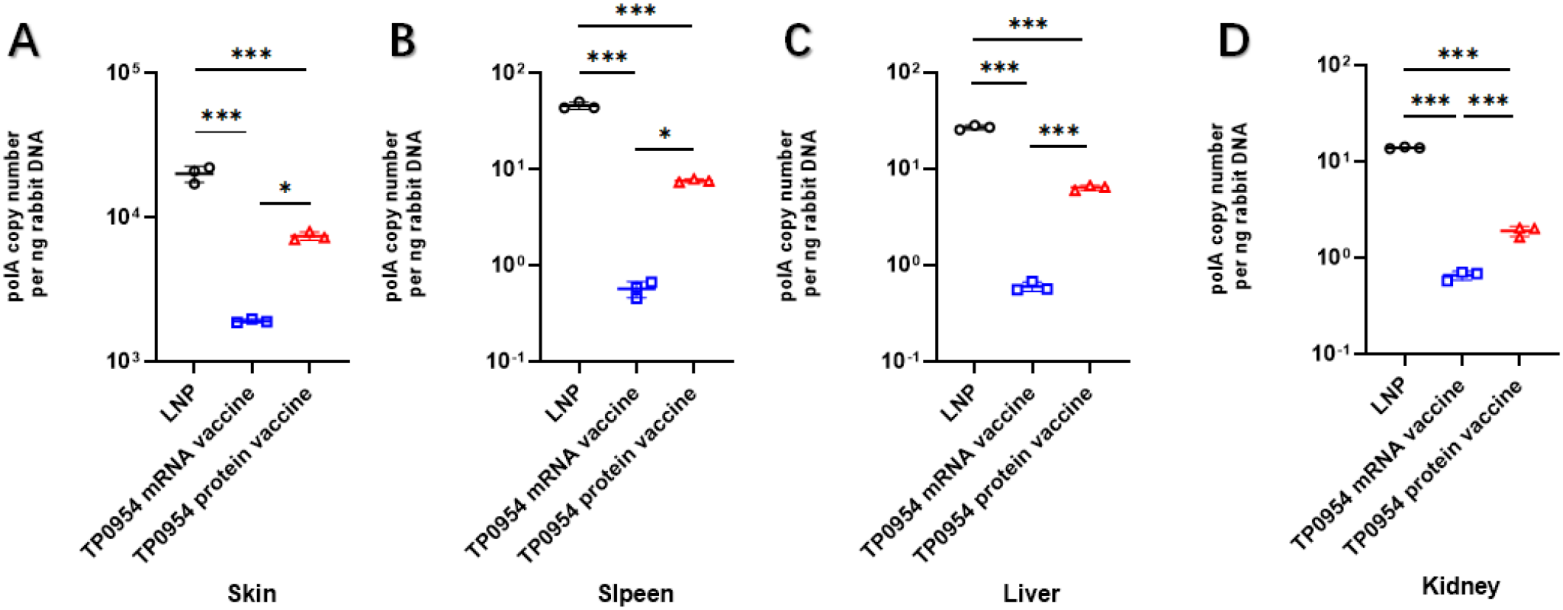
Immunization with TP0954 inhibited *T. pallidum* dissemination. The *T. pallidum* burden was evaluated in rabbits immunized with LNP (*N* = 3), TP0954 mRNA vaccine (*N* = 3) and TP0954 protein vaccine (*N* = 3) using quantitative real-time PCR to measure the *polA* DNA concentrations in the lesion biopsies **(A)** and distal organs including spleen **(B)**, liver **(C)** and kidney **(D)**. Results of each tissue type were normalized according to the concentration of rabbit gDNA. One-way ANOVA test was used to analyze the results. Repeatability analysis was performed on three tissue samples of each rabbit organ. Each dot represents the mean *polA* DNA concentration of three individually selective tissue samples from every organ of each rabbit. Horizontal lines represent mean values (ns, not significant. *P < 0.05, ***P < 0.001).

To evaluate the ability of mRNA vaccine immunization to prevent *T. pallidum* dissemination, all of the rabbits were killed at day 21 post-challenge. The total DNA was extracted from the cutaneous lesions at the initial infection sites as well as distal tissues or organs and qPCR was performed to determine *T. pallidum* DNA concentration to assess the treponemal burdens. As shown in **Figure 6**, all immunized rabbits showed decreased *T. pallidum* loads at cutaneous lesions. Moreover, The *T. pallidum* loads were significantly lower at cutaneous lesions in rabbits immunized with TP0954 mRNA vaccine compared with rabbits immunized with TP0954 protein vaccine. In addition, the *T. pallidum* burdens in the distal organs of the animals were also assessed (**Figure 6**). After immunization with TP0954 mRNA or protein vaccine, the burdens in the spleen, liver and kidney were also obviously decreased. And TP0954 mRNA vaccine immunization showed better efficiency in inhibiting *T. pallidum* dissemination compared with TP0954 protein vaccine. These results suggested that TP0954 mRNA vaccine prevented treponemal dissemination to distant organs, further confirming that TP0954 was a promising vaccine candidate.

### TP0954 mRNA vaccine immunization promoted the inflammatory infiltration

**Figure 7:**
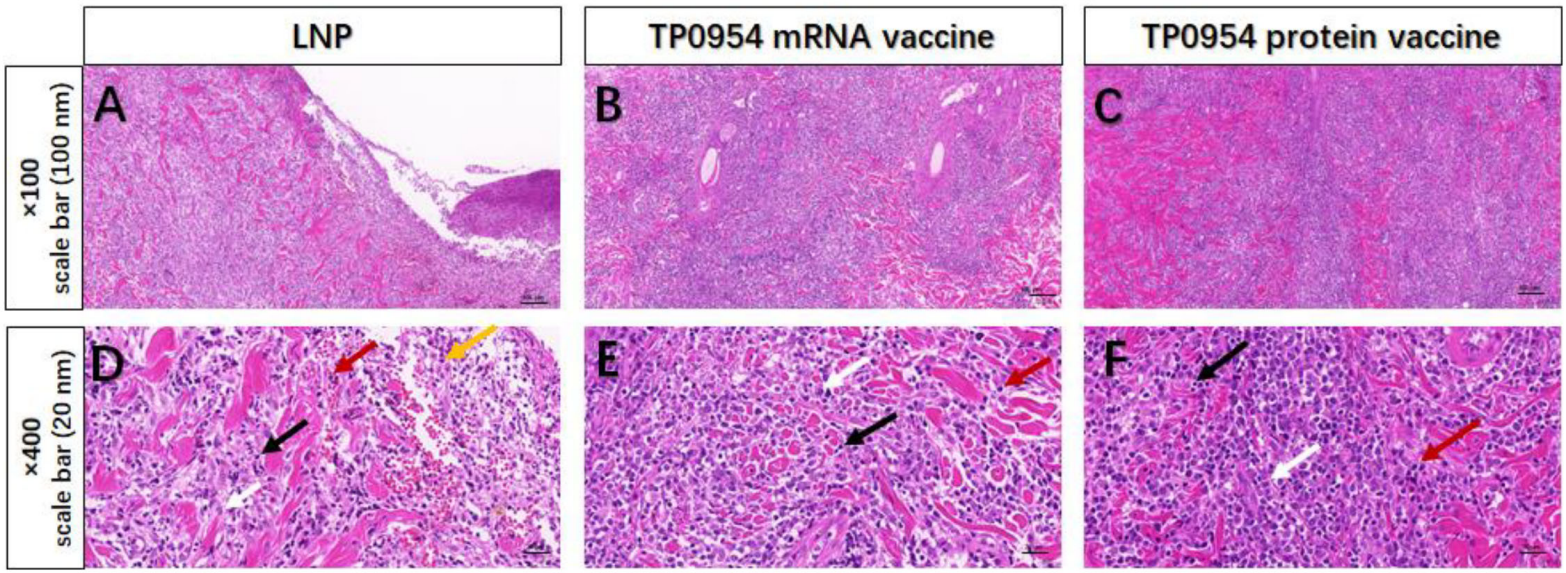
Histopathological analysis of primary cutaneous lesions on 21 days post-intradermal challenge with *T. pallidum*. On day 21 post *T. pallidum* challenge, cutaneous tissues from LNP group **(A, D)**, TP0954 mRNA vaccine group **(B, E)** and TP0954 protein vaccine **(C, F)** were sliced and stained with H&E. Primary cutaneous lesion sites of rabbits in each group showed inflammatory cell infiltration of varying degrees (black arrow =lymphocyte; red arrow = plasma cell; white arrow = macrophage; yellow arrow = neutrophil).

On day 21 post-challenge, H&E stained sections from primary cutaneous lesion sites of rabbits were performed to evaluate the intensities of inflammatory infiltration and cell types. All lesions revealed various degrees of infiltrates of inflammatory cells. In the lesions of LNP immunized rabbits, neutrophils, lymphocytes, plasma cells and macrophages were all observed. And compared with rabbits immunized with LNP, rabbits immunized with TP0954 mRNA vaccine and TP0954 protein vaccine had increased levels of lymphocytes, plasma cells, macrophages (**Figure 7**).

### The changes of serum TPPA and RPR titers in rabbits immunized with TP0954 mRNA vaccine

**Figure. 8:**
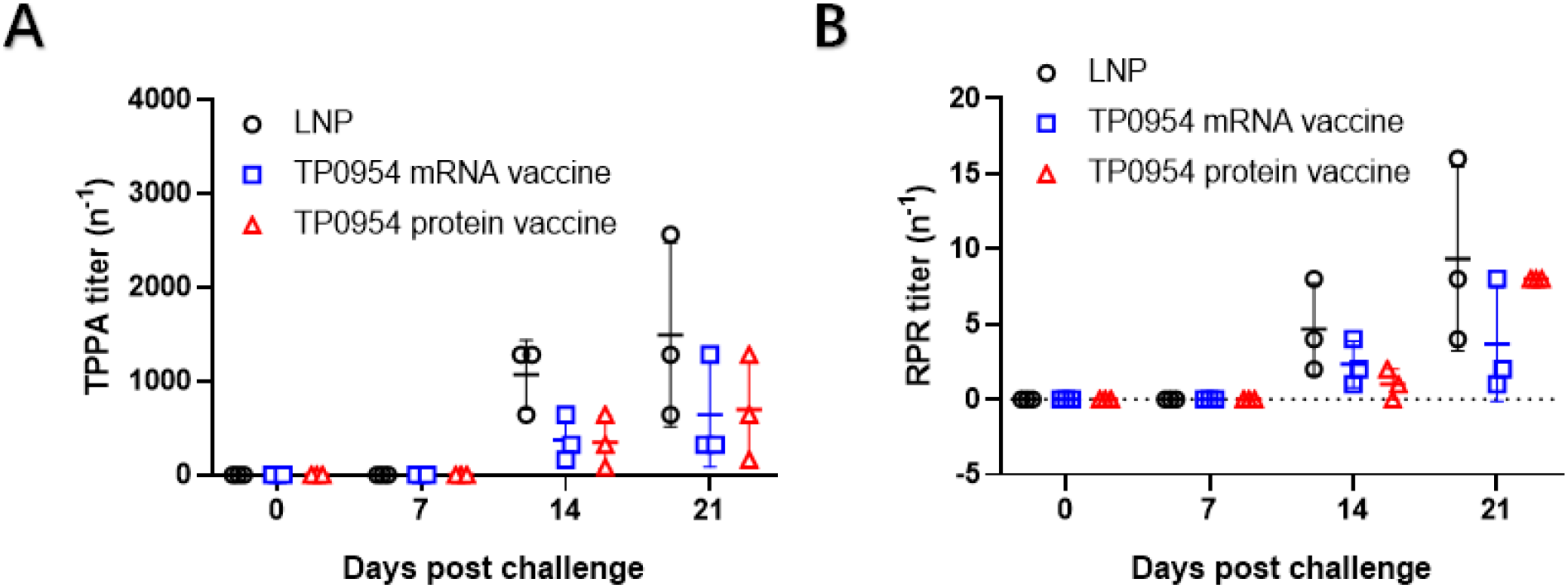
Serological changes post *T. pallidum* challenge. **(A)** TPPA titers were assessed on day 0, 7, 14 and 21 post *T. pallidum* challenge. **(B)** RPR titers were assessed on day 0, 7, 14 and 21 post *T. pallidum* challenge. One-way ANOVA test was used to analyze the results. Horizontal lines represent mean values.

As shown in **Figure 8**, the serum TPPA and RPR of all rabbits were negative on day 0 and 7 post challenge. The average titers of TP0954 mRNA and protein vaccine immunized rabbits were lower than those of LNP immunized rabbits on day 14 and 21. However, no statistical difference was observed probably due to the limited number of rabbits.

## Discussion

*T. pallidum* is generally classified as Gram-negative bacteria covered with the OM, periplasmic space with endoflagella, peptidoglycan layer, and inner membrane which surrounds a cytoplasmic cylinder (20). However, the composition of their OM differs remarkably from that of typical Gram-negative spiral-shaped bacteria. In addition to lacking lipopolysaccharides, the OM of *T*. *pallidum* has a relatively lower density of OMPs than Gram-negative bacteria such as *Escherichia coli* (approximately 1% of the number found in the *E. coli* OM), contributing to the poor immunogenicity of this pathogen (21). Multiple individual OMPs have been proved to be partially protective in animal models, including TP0136, TPRK and TP0954 (17, 22, 23). Although a single *T. pallidum* antigen could not induce complete protection, these studies provided essential candidates for developing the syphilis vaccines.

Previous studies suggested that TP0954 could be a conserved, immunogenic surface-exposed lipoprotein adhesion, mediating binding of spirochetes to the host epithelial, neuronal, and placental cells (24). TP0954 was also successfully expressed in *B. burgdorferi* B314 strain. Expression of TP0954 endowed the poorly adherent B314 strain with the capacity to bind to both epithelial and non-epithelial mammalian cell lines, a process mediated by its recognition of heparan sulfate and dermatan sulfate. Furthermore, TP0954 enhanced the binding of the B314 strain to placenta-derived cells and intact placental tissue. In addition, TP0954 displayed significant reactivity with sera obtained from syphilis patients and *T. pallidum* -infected rabbits across distinct stages of *T. pallidum* infection (25). This observation implied that TP0954 not only elicited a robust antibody response but was also constitutively expressed by *T. pallidum* during infection. All these findings implied that TP0954 could be a syphilis vaccine candidate to potentially inhibit transplacental transmission. Later, He et al (17) evaluated the immunogenicity and protection of recombinant Tp0954 in rabbits. The results showed the immunization with recombinant TP0954 induced antigen-specific humoral and T-cell immune responses, delayed the development of lesions and inhibited *T. pallidum* dissemination. Additionally, the rabbits receiving popliteal lymph nodes from TP0954-immunized, *T. pallidum*-challenged rabbits were not infected by *T. pallidum*, confirming sterile immunity. In this study, we constructed a novel mRNA vaccine targeting TP0954 and investigated if immunization with TP0954 mRNA vaccine was able to induce a strong and specific immune response and offer protection against *T. pallidum* infection. The results provided evidence that TP0954 mRNA vaccine elicited similar antigen-specific humoral response and stronger T-cell immune response compared with TP0954 protein vaccine. However, the serum TP0954 antibody level was lower in TP0954 mRNA vaccine group compared with TP0954 protein vaccine group. The lower antibody titers observed after primary immunization with the mRNA vaccine compared with the protein vaccine formulated with TiterMax Gold are not unexpected and can be explained by fundamental differences in antigen presentation kinetics and adjuvant mechanisms between the two vaccine platforms. Protein vaccines combined with strong water-in-oil adjuvants such as TiterMax Gold provide immediate and prolonged antigen availability at the injection site, which promotes rapid B-cell activation and robust early antibody production after the prime dose (26). In contrast, mRNA vaccines depend on *in vivo* translation of the encoded antigen, resulting in a delayed but more physiologically regulated antigen expression process. Consequently, primary antibody responses induced by mRNA vaccines are often lower than those elicited by protein vaccines formulated with highly potent experimental adjuvants. Importantly, after booster immunization, the antibody titers induced by the mRNA vaccine reached levels comparable to those elicited by the protein vaccine, indicating effective priming of immune memory. This characteristic immunization kinetics, modest primary responses followed by strong booster-dependent antibody amplification has been widely reported (11, 27–29). Moreover, it should be noted that TiterMax Gold is a highly potent adjuvant intended for experimental use only and is not approved for human vaccination. Therefore, the strong primary antibody response elicited by the protein vaccine represents an upper benchmark of antibody induction rather than a clinically translatable comparison. Overall, the comparable antibody titers observed after booster immunization support the conclusion that the mRNA vaccine elicits a robust and durable humoral immune response despite lower titers after the initial dose. Furthermore, compared to rabbits immunized with TP0954 protein vaccine, rabbits immunized with TP0954 mRNA vaccine was more effective in attenuating lesion development and decreasing treponemal burdens in lesions and distant organs. Therefore, our results further confirmed that TP0954 is a promising syphilis vaccine candidate and TP0954 mRNA vaccine showed better efficiency than protein vaccine.

mRNA-LNP vaccines have several advantages over protein vaccines formulated with strong experimental adjuvants such as Freund’ s adjuvant or TiterMax Gold adjuvant, particularly in terms of clinical translatability and safety. Firstly, mRNA-LNP is a clinically validated platform with multiple licensed human vaccines, demonstrating that the formulation and manufacturing pipeline can meet regulatory standards and be rapidly adapted to new antigens without requiring protein expression, purification or pathogen culture (30). Secondly, regarding safety and adverse effects, Freund’s complete adjuvant is explicitly considered unsuitable for human use due to marked reactogenicity and the risk of severe inflammatory lesions such as persistent injection-site inflammation and granulomatous reactions (31). This limitation is acknowledged in scientific guidance and reviews discussing adjuvant safety (32). Likewise, TiterMax Gold is intended for research use and is not for use in humans, making it an excellent tool for strong priming in animal experiments but not a clinically translatable comparator for human vaccine safety discussions (31). By contrast, mRNA-LNP vaccines typically cause mainly transient, self-limited reactogenicity such as local pain, fatigue, fever, consistent with innate immune activation, rather than the severe, persistent granulomatous pathology (33). In summary, compared with protein vaccines formulated with Freund’s adjuvant or TiterMax Gold, mRNA–LNP provides a more clinically relevant and regulatorily validated platform with better human-use suitability, while still achieving strong immune responses, and with side effects that are generally transient reactogenicity rather than severe, persistent local tissue damage seen with highly reactogenic experimental adjuvants.

Delayed-type hypersensitivity (DTH) reaction is critical for the efficient clearance of *T. pallidum* from lesion sites. This response relies on the local production of Th1 related cytokines especially interferon-γ (IFN-γ), which is secreted by CD4^+^ T cells, NK cells, CD8^+^ T cells, and resident dendritic cells. Subsequent activation of macrophages mediated by Th1 cytokines enhances the phagocytosis of opsonized *T. pallidum* (34). Therefore, Th1- related immune response is vital in preventing *T. pallidum* infection. We found the Th1 related cytokines secreted by spleen cells were elevated in rabbits immunized with TP0954 protein vaccine. And the levels were significantly higher in rabbits immunized with TP0954 mRNA vaccine. This result was consistent with the following *T. pallidum* challenge experiment. Both TP0954 mRNA vaccine and TP0954 protein vaccine exhibited faster immune responses against *T. pallidum* infection. Moreover, TP0954 mRNA vaccine showed better efficiency in attenuating lesion development and decreasing treponemal burdens in lesions and distant organs compared with TP0954 protein vaccine. Moreover, in the lesions of rabbits immunized with TP0954 mRNA vaccine and TP0954 protein vaccine, we observed increased level of macrophages, lymphocytes and plasma cells, which might play important roles in the DTH reaction. While substantial evidence supports the critical role of cell-mediated immunity in syphilis, it is well established that humoral immune response is equally essential. In animal models, neither passive immunization with humoral components nor adoptive T-cell transfer was sufficient to induce sterilizing immunity (34). Furthermore, opsonophagocytosis of *T. pallidum* by human or rabbit peritoneal macrophages depended on the presence of immune serum from patients or immune rabbit serum, respectively (34).

Although we did not observed a decline in serum TPPA and RPR titers in vaccinated rabbits after *T. pallidum* challenge, the vaccine efficacy is still meaningful. TPPA is a treponemal-specific assay that detects antibodies against *T. pallidum* antigens and is known to remain positive for prolonged periods regardless of bacterial clearance or treatment outcome (35). Therefore, TPPA titers are not considered a reliable marker for evaluating protective efficacy or bacterial elimination in vaccine studies. Similarly, RPR is a non-treponemal test that reflects host inflammatory responses to cellular damage rather than direct bacterial burden. RPR titers may remain stable or decline only slowly after infection or even after successful antimicrobial treatment (36). Consequently, changes in RPR titers do not necessarily correlate with reduced *T. pallidum* dissemination or lesion severity. Instead, vaccine efficacy should be evaluated using more appropriate endpoints, such as lesion progression, histopathological findings, bacterial burden assessed by PCR or dark-field microscopy, and cellular immune responses. These parameters provide a more accurate reflection of protective immunity against syphilis.

Despite being an early preclinical animal study, the findings offered meaningful translational implications for future clinical development. The integration of TP0954 mRNA vaccine into global syphilis control programs could be transformative, especially in low-income regions (1). mRNA vaccines are easier to scale, store, and distribute compared to traditional vaccines, which is an advantage in regions with limited cold-chain infrastructure (30). Moreover, the rapid production and adaptability of mRNA technology make it an ideal candidate for addressing emerging syphilis outbreaks. By complementing existing diagnostic and treatment programs, mRNA vaccines could be used as part of a broader strategy for syphilis prevention, reducing the incidence of congenital syphilis and enhancing overall reproductive health outcomes (37). By eliciting a robust immune response, the vaccine could slow down the development of syphilis, particularly in the early stages, thereby reducing the severity of disease and preventing the long-term complications associated with untreated syphilis, such as cardiovascular and neurological damage. This aspect could be particularly beneficial in populations at high risk of syphilis, providing an additional layer of protection even in the absence of complete immunity.

So far, this is the first study that successfully constructed TP0954 mRNA vaccine for *T. pallidum* and explored the immunogenicity and immune protection of mRNA vaccine in rabbit model of syphilis, filling the gap in research of mRNA vaccines for syphilis. Besides, TP0954 mRNA vaccine provided better protection against *T. pallidum* infection compared with TP0954 protein. Moreover, unlike TP0954 protein vaccine, TP0954 mRNA vaccine showed robust immune responses without additional adjuvants. Freund’s Previous adjuvant used in recombinant protein vaccines for syphilis such as Freund’s adjuvant could not be used in humans due to its side effects (38). Furthermore, a low dose of TP0954 mRNA vaccine could induce high level of immune responses and provide partial protection. Nevertheless, 5 µg dose used in this study should not be interpreted as a clinically optimized human dose, but rather as a biologically effective preclinical reference dose selected to enable comparison with protein-based vaccines in an established animal model. Importantly, evidence from licensed mRNA vaccines for other infectious diseases demonstrates that effective human doses are not fixed and may vary substantially depending on antigen properties, LNP formulation, immunization schedule, and host factors. For example, authorized SARS-CoV-2 mRNA vaccines employ doses ranging from 10 µg to 100 µg in adults, with lower doses shown to be sufficient in certain populations while higher doses increase reactogenicity without proportional immunogenic benefit (39). Inter-individual variability in optimal dosing is well recognized and may be influenced by age, sex, body composition, baseline immune status, and prior pathogen exposure (40). As a result, dose-ranging and regimen-optimization studies are a standard and essential component of early-phase clinical development for mRNA vaccines, rather than being determined directly from preclinical dose levels (30). Accordingly, the findings from the present study demonstrate that a low-dose of mRNA vaccine can elicit robust immunogenicity and protective signals in an experimental model, supporting the feasibility of the mRNA platform for syphilis vaccination.

There were also several limitations existing in this study. Systematic dose–response studies and formal cross-platform equivalency assessments were not performed in the rabbits immunization. The mass-based equivalence between protein and mRNA vaccines should be assumed in future work. Besides, TP0954 mRNA vaccine only showed partial rather than complete protection against *T. pallidum* Nichols strain. Meanwhile, The Nichols strain was selected for challenge experiments because it is a well-characterized reference strain with stable and reproducible virulence in the rabbit model, is readily maintained under laboratory conditions, and remains relevant for vaccine evaluation given the high conservation of key candidate antigens. However, the other strains such as MexicoA and SS14/Sea81–4 strains were not included in this study and should be investigated in the future work. This study mainly focused on Th1-related cellular responses. Other potential protective mechanisms were less explored. Future research will be conducted to further explore immune pathways beyond Th1 responses. Moreover, this study examined the immune protection only four weeks after the primary immunization, lacking further long-term observation. Therefore, long-term immunity should also be tested both in immunized mice and rabbits. Finally, the statistical power was limited by the small number of animals used in the experiments.

## Conclusion

In conclusion, our results further confirmed that TP0954, a key adhesion protein of *T. pallidum*, represented a promising vaccine candidate. Immunization with TP0954 mRNA vaccine could induce antigen-specific humoral and T-cell immune responses. Moreover, rabbits immunized with TP0954 mRNA vaccine showed better efficiency in attenuating lesion development and reducing treponemal dissemination compared with TP0954 protein vaccine. Even if the vaccine could not fully prevent infection, it might prevent the disease from progressing to secondary or tertiary stages. Future studies should consider the development of a multi-component vaccine containing more than two protective mRNA vaccines. With the continuous advancement of technologies, there were several studies reporting that *T. pallidum* could be cultured *in vitro* and genetic manipulation of this pathogen was achievable, providing new insights for syphilis vaccines research.

## Data Availability

The original contributions presented in the study are included in the article/**Supplementary Material**. Further inquiries can be directed to the corresponding authors.

## Supplementary material

Supplementary Table 1 The sequence of TP0954 mRNA.

Supplementary Figure 1 Lesions on the backs of the rabbits on 21 days post-intradermal challenge with *T. pallidum* (high-definition, un-magnified and whole-field original image of the skin).
