# Supplementary material for: Treponema pallidum mRNA-LNP vaccine candidate encoding TP0954 induces strongly protective immunity in rabbits
Source: Front Immunol. 2026 Feb 18;17:1769155. doi: 10.3389/fimmu.2026.1769155 (PMC12957224; doi:10.3389/fimmu.2026.1769155)

Supplemantary table 1. The sequence of TP0954 mRNA.

| 5’UTR |
| --- |
| UAAUACGACUCACUAUAAGGAGAAUAAACUAGUAUUCUUCUGGUCCCCACAGACUCAGAGAGAACCCGCCACC |
| TP0954 CDS |
| AUGCGGUAUGGCACUCUUUUUAAGGUGAGUGUGCUCUUAGGUGCGCUCUUUGUAUCUUGUGUUAGUACUGGAUCGAACAGCGCUCGGGAGAGCGAGCGGGCACAGCUUUUAAAAAGCGAAAAUCCUAAUAUCCGUUUUGCAGCUCAGCUCAGUGGGUUGUUGGAAAAGCAGCGGUGGGAAGAGGCGUUGCAGCUGUUCGAUACGUUGAGCCCUGAGCAUCGUGCCGAAAAGCGCAUCCAAUACCUGUAUCUUUCUACGCUCAUUUCCGCAGGGAAAUUGACGCACGCGCAGGAGCUUGCAGAAAAGCUUGGGCAGGACGGUCCUACGGCUUCUGAAACGGUGCAACUGUGGUACGCACACGCAAUGAUAGCGCAAGCGAAACGAGAUGUGCGCAAGAAGAAACAGUAUGUAGAAAAGAUUCUUGCGCAGGAUCCGCACGAUCUGUGGGCACUGACUGAACGCGGUUACGAUUUUUUAAGUGUUAAUGAUUAUGCCCAGGCGGUGCAGGCUUUCUCGCGCGCGCUGCGCGUGGAGCCGCGUGCGCAGGACGCGCGGGUAGGUUUGGGGAAGGUGUACUAUCUGCAGGGAAAGAUGCAAGAGGCAGAGGCACAGUACCGACAGGUACUGCAGGAUACGCCUGAACAUGAGCGCGCGCUUGCAGAAUGUGCGCGGGUGAAAGCGGAAACGAACCGAGUGCUCGAAGCUAUUAGGGAUCUGGAACGUGUGGUGCAGCUUGAUCCGCAUGAUCCUGCAUAUUGGACAGAUCUCGGCACGUAUUUGUCCCAGGCGGGAAAAAAGGAGCGUGCGGCAGCGGCGUUCGAGCGCGCAGUGGCGCUUUCUGCUGAUGCGUAUUUUGCCCACAUUUAUCUGGGGGGAAUUUACGACGAGCUCGGGCGAGCGGAGAAAGCGAUUGAGCACUAUCAGCGCGCGGUGCAGCUGUAUCCGAAAUACCAUUUUUCUUUCGAAAGCUUAGGAGUGCUAUUUUGGGAGCAGCAGCGGUGGGAAGAGGCGCGUGAGGCUUUUGCAACUGCGCUUACCUACGCCCCAACGAAUAUCUCGUAUGCGCUCAUGACGGCGCUGUGUUUGUGUCAGAUGGGCCAGGCACACAAGGCGCAGCACUUUAUGCGGACGUUUAUUCGGACCGUAGAUAGAACGCGACGGGAAGUGGAGUAUUUUUUGUGUCGCUUGUUUGUCGAUUUGAGUGGGGAACAGGAUAUGGCUUCCCGUAUAUCUAAAAUUAAGUCAGUGCCCCAGCGUAUAAGAUACUCGUUUUACCUUGCCUUUUUUUACGAACUCGGGGGGCGGCAUCUGCUUGCCGAGAAACAUUACGGUGAAGUAGAAUCGGCGCGUGCUCCUUCUUCCUUUGAGCACCGACUUGCGGUGAGUGCUCUUGGGCGUUUACGAGGGAGGUCAUCGUCGCUGCGUACGAAUCCCUAG |
| 3’UTR |
| UAACUCGAGCUGGUACUGCAUGCACGCAAUGCUAGCUGCCCCUUUCCCGUCCUGGGUACCCCGAGUCUCCCCCGACCUCGGGUCCCAGGUAUGCUCCCACCUCCACCUGCCCCACUCACCACCUCUGCUAGUUCCAGACACCUCCCAAGCACGCAGCAAUGCAGCUCAAAACGCUUAGCCUAGCCACACCCCCACGGGAAACAGCAGUGAUUAACCUUUAGCAAUAAACGAAAGUUUAACUAAGCUAUACUAACCCCAGGGUUGGUCAAUUUCGUGCCAGCCACACCCUGGAGCUAGC |
| Poly(A) tail |
| AAAAAAAAAAA |

Supplemantary figure 1. Lesions on the backs of the rabbits on 21 days post-intradermal challenge with *T. pallidum* (high-definition, un-magnified and whole-field original image of the skin).


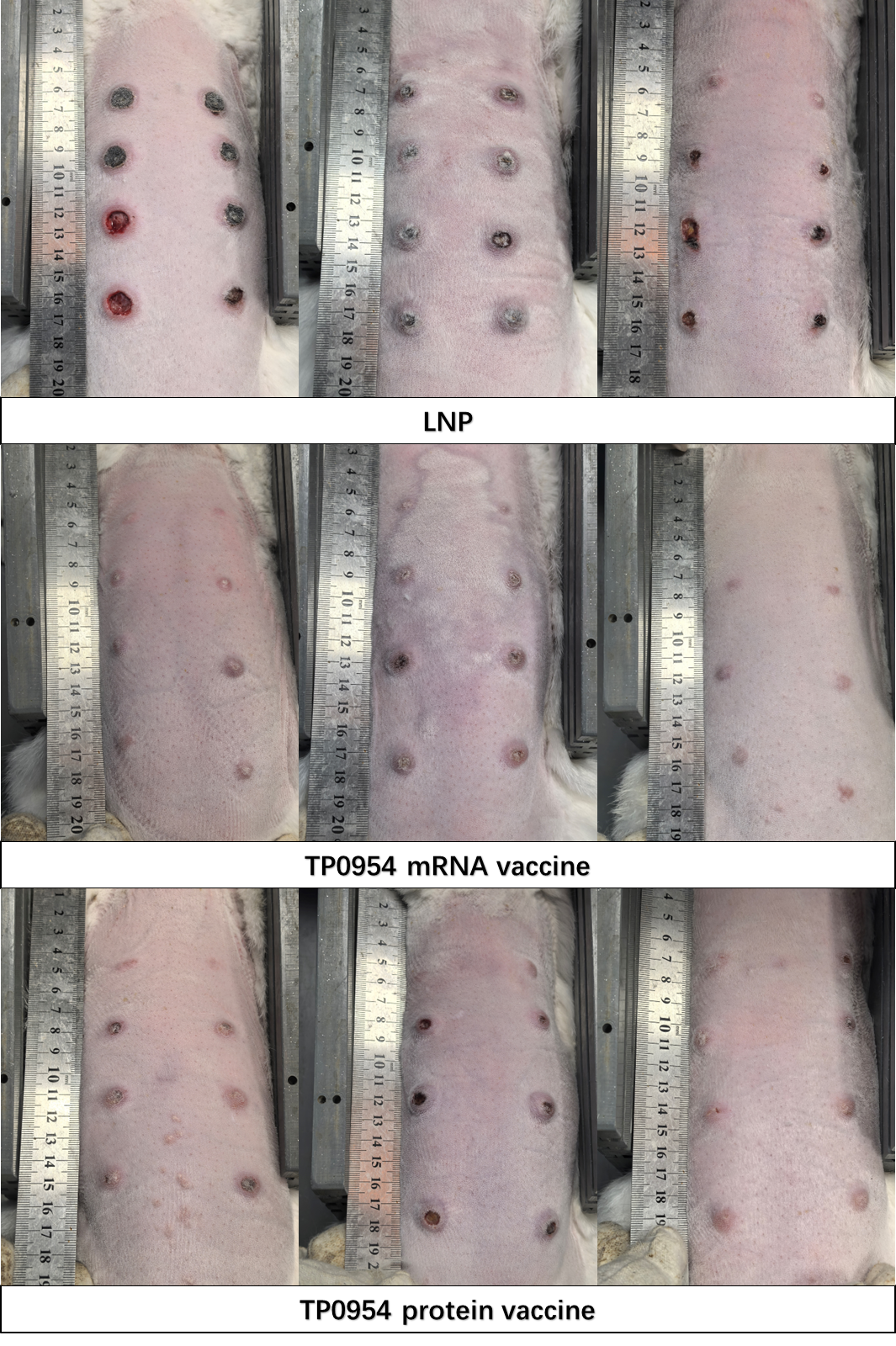

Supplement: Supplementary Table 1 — The sequence of TP0954 mRNA. [file Table1.docx]
